# Conservative Management of Combined Pleural and Splenic Injury During Percutaneous Nephrostolithotomy

**DOI:** 10.1089/cren.2016.0107

**Published:** 2016-11-01

**Authors:** Geoffrey S. Gaunay, Haris Ahmed, Arthur Smith, Zeph Okeke

**Affiliations:** Department of Urology, The Smith Institute for Urology, Northwell Health, Lake Success, New York.

**Keywords:** nephrolithiasis, percutaneous nephrostolithotomy, PCNL complications, renal calculus, splenic injury

## Abstract

Splenic injuries related to percutaneous nephrostolithotomy (PCNL) are infrequent. Herein, we report a combined splenic and pleural injury incurred during PCNL along with radiographic images documenting the complication. A review of management techniques for similar injuries is included.

## Introduction

Percutaneous nephrostolithotomy (PCNL) is the treatment of choice in symptomatic patients with renal stone burdens >20 mm in size. Although safe and efficacious, complications are not infrequent, the vast majority (95.1%) of which are minor in character (Clavien Grades I & II).^1^

Although well described, pleural injuries are relatively infrequent, observed in <2% of cases.^2^ PCNL-related pleural injury can result in pneumothorax, hydrothorax, hemothorax, or urinothorax, leading to delays in recovery and potentially requiring additional therapeutic interventions. Intraperitoneal organ injury as a result of PCNL access and/or dilation is exceedingly rare (0%–1.7%), most commonly involving colonic perforation.^1,3^ Particularly in left-sided PCNL, injury or rupture of the spleen is a feared complication, potentially resulting in devastating intraperitoneal hemorrhage. As it is described in only a handful of cases in the literature, optimal management of PCNL-related splenic injuries remains unclear.

To better illustrate the respective management of pleural and splenic injuries, we present a case of a left PCNL complicated by pneumothorax and trans-splenic access. To our knowledge, this is the first case of combined pleural and splenic injury described in the literature.

## Case Report

**FIG. 1.**
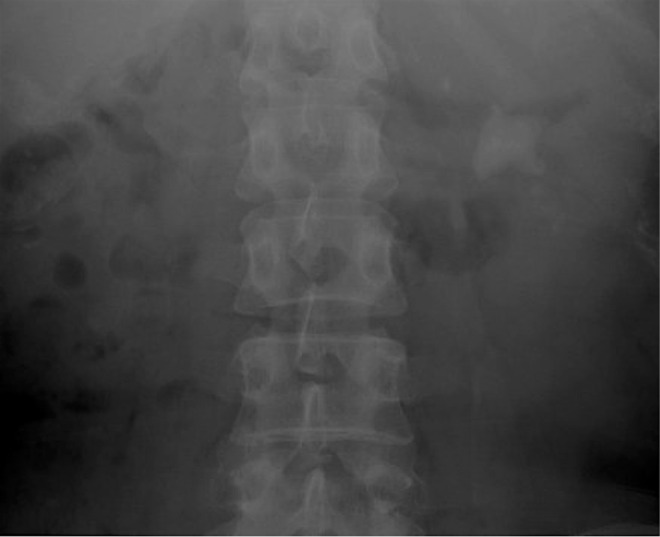
Preoperative abdominal radiograph showing large left-sided partial staghorn calculus.

An otherwise healthy 45-year-old Asian female was referred to our institution after hematuria evaluation revealed a leftsided partial staghorn calculus encompassing the central region and lower pole calices (Fig. 1). Stone size precluded ureteroscopic or extracorporeal treatment methods, and PCNL was recommended.

**FIG. 2.**
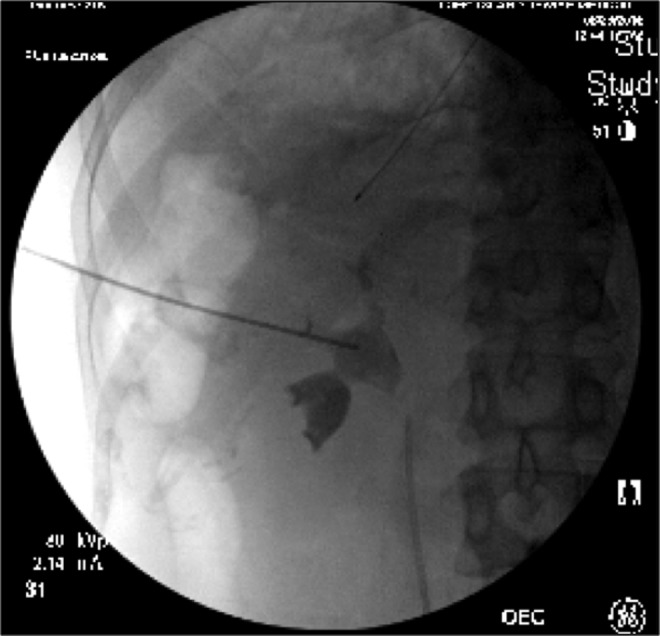
Intraoperative fluoroscopic image showing left interpolar renal access.

In the operating theatre while in the prone position, interpolar renal access was achieved above the level of the 12th rib (Fig. 2). Balloon dilatation of the tract was performed and a 30F sheath was introduced as a working channel. Uneventful rigid nephroscopy with ultrasonic lithotripsy followed by flexible nephroscopy was performed with complete stone removal. A re-entry Malecot nephrostomy catheter was introduced for postoperative drainage. A ureteral stent was not placed. Nephrostogram at the conclusion of the procedure revealed no significant extravasation and prompt transit of contrast down to the level of the bladder. There were no hemodynamic or respiratory issues throughout the procedure and estimated blood loss throughout was ∼100 mL.

**FIG. 3.**
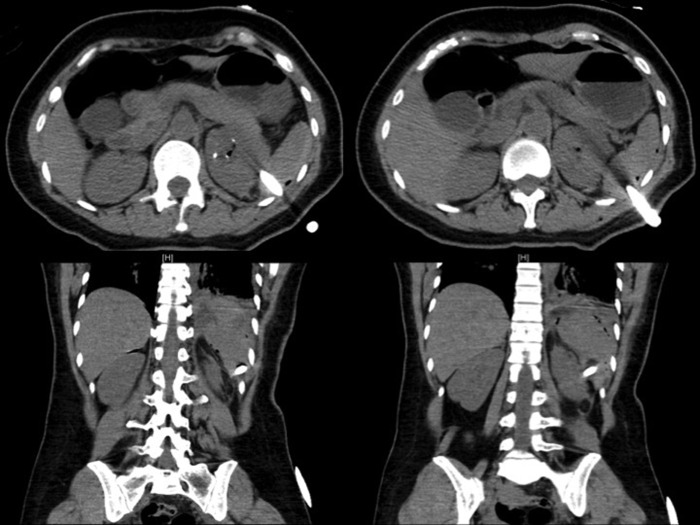
Axial and coronal computed tomography images showing trans-splenic nephrostomy catheter.

**FIG. 4.**
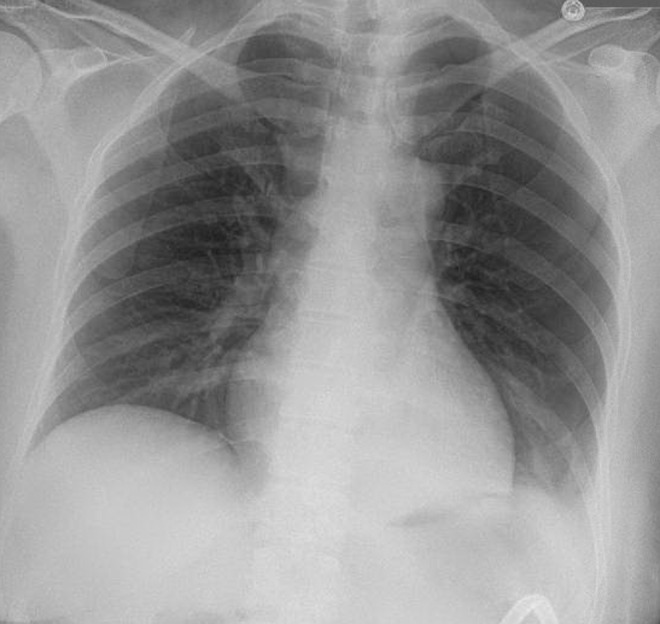
Postoperative day 1 chest radiograph reveals large left pneumothorax.

Chest radiograph obtained in the recovery room was normal 1 hour after surgery. Postoperative hematocrit declined to 33.0 from 41.3 before surgery; however, vital signs were within normal limits. A noncontrast computed tomography (CT) scan was performed on postoperative day (POD) 1, per our institutional protocol, showing a large left pneumothorax and nephrostomy catheter traversing the spleen (Fig. 3). Subsequent radiograph confirmed the pulmonary findings, showing a large left pneumothorax (Fig. 4). A small perinephric hematoma was also noted; however, no significant intraperitoneal bleed was identified. Pulmonary consultants placed a thoracostomy drainage catheter in the left pleural space. Resolution of the pneumothorax was noted on subsequent imaging.

After chest decompression, the patient had no significant complaints. Interval laboratory values and vital signs were all within normal limits and urinary output showed no sign of gross hematuria. The decision was made to proceed with conservation, nonoperative management of the splenic injury.

The patient was observed with serial abdominal examinations and laboratory monitoring. The re-entry catheter was removed at the bedside on POD 6 that was tolerated well by the patient. In advance of planned nephrostomy catheter removal, the patient was kept without food or drink after midnight (NPO) should surgical intervention be necessary. However, the patient's vital signs, laboratory values, and physical examination remained unchanged. Delayed chest tube removal was planned to determine whether nephrostomy removal would produce a recurrence of the left pneumothorax. Once stability of chest imaging was determined, the pigtail catheter was clamped for 24 hours and subsequently removed on POD 7 after chest radiograph showed no evidence of pneumothorax. The patient was discharged the following day. Follow-up visit on POD 15 demonstrated an unremarkable physical examination and stability in hematocrit.

## Discussion

Relative to the sheer number of PCNL procedures performed annually around the world, splenic injury is fortunately a rare occurrence in less than 0.5% of procedures.^4^ There have been no large series descriptions of PCNL-related splenic injuries. Indeed, only 11 such cases were described in a recent review of gastrointestinal complications of PCNL. To our knowledge, this is the first such complication at our institution. However, as splenic injury is most commonly identified on postoperative imaging, which is not regularly obtained, cases are likely underreported. However, cases of splenic hemorrhage presenting at the time of nephrostomy removal have been described.

Access to the superior pole of the left kidney poses the greatest risk to splenic injury, particularly in those with retrorenal spleens.^3^ Access within the 10th or 11th intercostal spaces drastically increases the likelihood of splenic injury. In addition, risk may vary based on the inspiratory/expiratory phase. On average, the greatest risk occurs with access above the 11th or 12th ribs during inspiration: up to 33%.^5^ Lower pole access may have avoided this complication in our patient; however, the benefits of such must be weighed against the potential access-related difficulties of stone manipulation.

Debate over the superiority of supine versus prone PCNL continues. Upper pole access in the supine position increases the likelihood of splenic injury versus similar prone access, as the spleen is more medially situated.^6^ Anatomical studies utilizing both prone and supine CT to assess for potential access-related organ injury found a 7% risk of splenic injury with upper pole access in the supine position versus a 0% risk in the prone position.^7^ Although supine renal access may have greater propensity for splenic injury, thorough review of preoperative imaging to ascertain the location of adjacent organs and assess for anatomic variability is recommended regardless of patient positioning.

Unfortunately, there exists no consensus on the proper management of PCNL-related splenic injury, although a number of effective methods have been employed.

Given the potential for intraperitoneal hemorrhage, some advocate immediate exploratory laparotomy with splenectomy after splenic injuries are identified.^4^ A subset of prior reported cases have been managed in this manner, and certainly those patients with hemodynamic instability should undergo an exploratory procedure. However, optimal management of hemodynamically stable patients is unclear.

The trauma literature provides more extensive experience with splenic injuries and guidelines on their management. The Eastern Association for the Surgery of Trauma (EAST) Guidelines recommend against routine laparotomy in hemodynamically stable patients with isolated splenic injuries, regardless of the severity of the injury.^8^ Guidelines also consider angiography with embolization in those hemodynamically stable patients with an American Association for the Surgery of Trauma (AAST) grade III or greater lesion, moderate hemoperitoneum, or those with evidence of ongoing bleeding.^8^ Non-operative management (NOM) of penetrating splenic injury is applicable in patients presenting without hemodynamic instability; however, up to 20% may fail within 24 hours because of associated hollow viscus organ injury.^9^

Conservative management of PCNL-related splenic injury has been described in several case reports.^3^ Most management methodologies consisted of delayed nephrostomy tube removal, to tamponade bleeding sources and allow for track maturation.^3^ The most appropriate time for nephrostomy removal is unclear; however, the tube should be maintained should signs or symptoms of hemorrhage be present. We elected for nephrostomy removal on POD 6 after several days of close observation. Other catheter durations in case series have ranged from 4 to 15 days.^3^

Several cases detail the injection of thrombostatic agents into the nephrostomy track. Desai et al. reviewed a similar complication managed by concurrent ureteral stent and trans-splenic percutaneously introduced Gelfoam^®^ pledgets on POD 2.^10^ Similarly, Thomas described a technique wherein a collagen–thrombin hemostatic sealant (D-Stat; Vascular Solutions, Inc., Minneapolis, MN) was introduced through the nephrostomy tube during removal in the interventional radiology suite.^11^ Both patients had uneventful recovery periods.

Pleural injury is more common in supracostal renal accesses, in up to 16% of patients.^1^ Accesses in the 10th intercostal space is associated with a higher likelihood of pleural injury within the 11th intercostal space (35% *vs* 10%).^1^ Again, thorough review of preoperative imaging is essential to understand anatomic relationships between access points and the pleura. Pleural complications may be minimized by avoidance of supracostal access. When necessary, supracostal puncture should be performed during maximal expiration as the diaphragm and pleura are in the most cephalad position.^1^

Post-PCNL pneumothoraces may be managed expectantly, as many resolve without additional treatment. Chest radiography may be serially assessed for resolution. In large or symptomatic cases, thoracostomy tube or catheter placement is advised. Our patient exhibited a large pneumothorax with shortness of breath necessitating pigtail catheter placement. Although follow-up chest imaging showed prompt resolution of the pneumothorax, we elected to maintain the pleural catheter until removal of the nephrostomy. The rationale being that with removal of the tamponade generated by the nephrostomy catheter, a defect in the pleura may allow recurrence of the pneumothorax, requiring maintenance of the pleural drain.

Combined pleural and splenic injuries during PCNL are serious complications, which may be managed conservatively in appropriately selected patients. Although NOM of splenic injury during PCNL does have potential for significant hemorrhage at the time of nephrostomy removal, when successful spares the patient from a morbid exploratory procedure and splenectomy. Care should be directed toward monitoring for hemodynamic instability or laboratory evidence of hemorrhage when NOM is planned.

## Abbreviations Used

AAST: American Association for the Surgery of Trauma
CT: computed tomography
EAST: Eastern Association for the Surgery of Trauma
PCNL: percutaneous nephrostolithotomy
POD: postoperative day
